# Health-Related Social Needs in Patients With Gastroparesis: Relationships to Symptom Severity and Quality of Life

**DOI:** 10.1016/j.gastha.2023.09.001

**Published:** 2023-09-06

**Authors:** Susie O. Lee, Alexandra C. Barrett, Paul J. Silver, Henry P. Parkman

**Affiliations:** Division of Gastroenterology, Lewis Katz School of Medicine at Temple University, Philadelphia, Pennsylvania

**Keywords:** Gastroparesis, Health-Related Social Needs, Quality of Life

## Abstract

**Background and Aims:**

Patients with health-related social needs (HRSNs) experience barriers to health care services. To identify areas of intervention, we need to understand the impact of HRSN in patients with gastroparesis. This study aimed to 1) determine types of HRSN present in patients with gastroparesis; 2) analyze relationship between HRSN and gastroparesis symptom severity and health-related quality of life (HRQL); and 3) evaluate which HRSN domains most significantly affect symptom severity and HRQL.

**Methods:**

Patients with gastroparesis were enrolled and completed questionnaires to assess the following: 1) severity of gastroparetic symptoms using Gastroparesis Cardinal Symptom Index (GCSI); 2) HRSN using screening questionnaire; and 3) HRQL using the Patient Assessment of Upper Gastrointestinal Disorders-Quality of Life (PAGI-QOL).

**Results:**

Three hundred twenty-one patients with gastroparesis participated in this study. Two hundred twelve patients completed GCSI and HRSN questionnaires, and 109 additional patients completed PAGI-QOL questionnaire. Of the 321 total patients, the most common HRSN were mental health, financial strain, and food insecurity. Overall, 43% had at least one HRSN and 22% had at least 2 HRSN. The number of HRSN was directly correlated to the GCSI total symptom score (r = 0.284, *P* < .05) while inversely correlated to the PAGI-QOL score (r = −0.650, *P* < .05). Of the 7 HRSN domains studied, patients with mental health HRSN, in particular, reported more severe gastroparesis symptoms and lower quality of life.

**Conclusion:**

A large number (43%) of patients with gastroparesis had at least 1 HRSN. Patients with HRSN reported more severe gastroparesis symptoms and lower quality of life than patients without HRSN.

## Introduction

Gastroparesis is a syndrome characterized by delayed stomach emptying.^1^ Symptoms of gastroparesis include nausea, vomiting, early satiety, postprandial fullness, and abdominal pain. It is often associated with underlying conditions such as diabetes mellitus. Treatment of gastroparesis involves dietary modifications, medicines such as prokinetic agents to enhance gastric emptying, and antiemetic agents to reduce nausea and vomiting. In patients with severe symptoms, surgical intervention may be needed. There are also patients with symptoms of gastroparesis but with normal gastric emptying; these patients have similar demographics to patients with gastroparesis.^2^

Social determinants of health (SDOH) are the conditions in which people are born, grow, work, live, and age and also include the wider set of forces and systems shaping the conditions of daily life. Social determinants of health (SDOH) can include income and social protection, education, job security, early childhood development, and access to affordable high-quality health-care services. Health-related social needs (HRSNs), on the other hand, are the needs of an individual as a result of SDOH.^3^ Housing instability and food insecurity are associated with delayed medical care, poor access to ambulatory care, and increased emergency department visits.^4^^,^^5^ As a result, HRSN is increasingly the focus of interventions within health care.

Treatment of gastroparesis and its underlying conditions requires patients to have proper access to health care. However, patients with HRSN—including housing instability, lack of transportation, and food insecurity—often experience barriers to accessing health care services. These barriers can exacerbate existing health conditions and cause worse overall health outcomes. Studies have shown the impact of SDOH on patient outcomes for other disease processes. Patients with greater needs within SDOH domains showed increased risk of fatal coronary heart disease and nonfatal myocardial infarction.^6^ Adverse SDOH in rural patients are associated with a lower overall survival compared to urban patients without adverse social determinants of health.^7^ The impact of HRSN on patient outcomes remains to be studied.

The purpose of this study is to gain a better understanding of HRSN in patients with gastroparesis, assess the association between HRSN and severity of symptoms, and assess which HRSN domains most significantly affect the severity of symptoms and response to treatment in patients with gastroparesis. We will also study the association between HRSN and quality of life by using the Patient Assessment of Upper Gastrointestinal Disorders Quality of Life.^8^ We will measure severity of symptoms in patients diagnosed with gastroparesis using the Gastroparesis Cardinal Symptom Index.^9^ We intend to explore patients’ health-related social needs through a questionnaire adapted from the HRSN screening tool created by the Centers for Medicare and Medicaid Services (CMS) Accountable Health Communities Model.^10^ We propose that greater HRSN will correlate with an increased severity of symptoms, more impaired quality of life, and poorer response to treatment.

## Methods

### Patients

All patients 18 or older with gastroparesis who were established within the Temple University Hospital GI Section being seen in a follow-up clinical appointment were asked to participate. Patients who are pregnant, imprisoned, or cognitively impaired; who do not understand or speak English; or adults unable to consent were excluded from the study. If patients wish to participate, they signed an informed consent and authorization form for participation in this study.

### Gastroparesis Cardinal Symptom Index

The severity of gastroparesis symptoms was measured by using GCSI which quantifies the severity of 9 symptoms over the prior 2 weeks: nausea, retching, vomiting, stomach fullness, inability to finish a meal, excessive fullness, loss of appetite, bloating, and abdominal distension. Each of these 9 symptoms were divided into 3 subscores of nausea/vomiting (nausea, retching, vomiting), fullness/early satiety (stomach fullness, inability to finish a meal, excessive fullness, loss of appetite), and bloating (bloating, abdominal distension). The severity of each gastroparetic symptom was quantified using a 6-point Likert response scale which ranged from 0 (none) to 5 (very severe). The total GCSI score was calculated as the mean of the scores of the nausea/vomiting, fullness/early satiety, and bloating subscores.

### Health-Related Social Needs Questionnaire

Patients filled out a questionnaire on HRSN which was adapted from the CMS’ Accountable Health Communities Health-Related Social Needs Screening Tool.^11^ This questionnaire explores a patient’s HRSN in 7 domains: living situation, food security, access to transportation, utilities, interpersonal safety, financial strain, and mental health. Patients’ needs in each domain were determined based on the scoring guideline provided by CMS’ screening tool. The total HRSN score was calculated by adding up the score of all 7 domains. Scores could range from 0 to 7 with lower scores indicating lesser needs and higher scores indicating more needs.

### Patient Assessment of Upper Gastrointestinal-Quality of Life (PAGI-QOL)

The PAGI-QOL is an assessment tool which measures the quality of life of patients with upper GI disorders. It consists of 30 items divided into 5 subcategories: daily activities (10 items), clothing (2 items), diet and food habits (7 items), relationship (3 items), and psychological well-being and distress (8 items). Each item was quantified based on a 6-point Likert scale which ranged from all of the time (0) to none of the time.^5^ The total score was calculated by taking the mean of the subcategory scores. Total score could range from 0 to 5 with lower scores indicating poorer quality of life and higher scores indicating better quality of life.

### Data Analysis

Data from questionnaires were entered into a deidentified Excel spreadsheet. We analyzed the association between HRSN and severity of symptoms and the association between HRSN and quality of life by using a t-test for samples assuming unequal variances. We correlated HRSN scores against GCSI and against PAGI-QOL using Pearson correlations as weak (0.00–0.30), moderate (0.31–0.50), or strong (0.51–1.0) correlations, with significance defined at <0.05. In the final part of our study, we compared the average GCSI scores between patients with and without HRSN in each domain using a t-test for samples assuming unequal variances, with significance defined at <0.05.

## Results

Three hundred twenty-one patients with gastroparesis participated (262 females, 59 males; average age 44.4 ± 15.2 years; 94 diabetic, 199 idiopathic, 2 postsurgical) (Table 1). Two hundred twelve patients completed GCSI and HRSN questionnaires; subsequently, 109 additional patients also completed with PAGI-QOL questionnaire. Thirty-eight patients (11.8%) had GCSI scores between 0 and 0.99, 71 patients (22.1%) had GCSI scores between 1 and 1.99, 87 patients (27.1%) had GCSI scores between 2 and 2.99, 85 patients (26.5%) had GCSI scores between 3 and 3.99, and 40 patients (12.5%) had GCSI scores between 4 and 4.99 (Table 1).

**Table 1:** Demographic Information, Range of GCSI, Number of HRSN’s, and Range of PAGI-QOL

Demographics	n (HRSN) = 321	Percentage
	n (PAGI-QOL) = 109	
Age (Mean)	44.41 (±15.20)	
Sex:		
Male	59	18.4%
Female	262	81.6%
Race/Ethnicity:		
Caucasian	248	77.3%
African American	36	11.2%
Hispanic	19	5.9%
Asian American Pacific Islander	2	0.6%
American Indian	3	0.9%
Multiracial	5	1.6%
Other	8	2.5%
GCSI:		
0–0.99	38	11.8%
1–1.99	71	22.1%
2–2.99	87	27.1%
3–3.99	85	26.5%
4–4.99	40	12.5%
5	0	0%
Number of HRSN:		
0	183	57%
1	66	20.6%
2	33	10.3%
3	22	6.9%
4	9	2.8%
5	5	1.6%
6	3	0.9%
7	0	0%
PAGI-QOL:		
0–0.99	3	2.8%
1–1.99	14	12.8%
2–2.99	21	19.3%
3–3.99	31	28.4%
4–4.99	38	34.9%
5	2	1.8%

### Range and Types of HRSN of Patients with Gastroparesis

Of the 321 total patients, the most common HRSN domains were mental health (81 patients), financial strain (75 patients), food insecurity (47 patients), and transportation needs (30 patients) (Table 2). Only 8 patients (2.5%) with GCSI scores between 0 and 0.99 had needs in at least 1 HRSN domain compared to 51 patients (15.9%) with GCSI scores between 3 and 3.99. Overall, 138 patients (43%) had needs in at least 1 HRSN domain, 72 patients (22%) had needs in at least 2 HRSN domains, and 39 patients (12.1%) had needs in at least 3 HRSN domains.

**Table 2:** Prevalence of HRSN Among Gastroparesis Patients With Varying Severity of Gastroparesis Symptoms via GCSI Scores and Varying Quality of Life via PAGI-QOL

GCSI/PAGI-QOL scores	Living situation	Food insecurity	Transport needs	Utility needs	Interpersonal safety	Financial strain	Mental health
GCSI	n = 19	n = 47	n = 30	n = 15	n = 7	n = 75	n = 81
0–0.99	1 (5%)	1 (2%)	1 (3%)	1 (7%)	1 (14%)	6 (8%)	2 (2%)
1–1.99	4 (21%)	10 (21%)	4 (13%)	0 (0%)	0 (0%)	11 (15%)	10 (12%)
2–2.99	2 (11%)	6 (13%)	6 (20%)	4 (27%)	1 (14%)	16 (21%)	19 (23%)
3–3.99	7 (37%)	20 (43%)	13 (43%)	5 (33%)	5 (71%)	29 (39%)	35 (43%)
4–4.99	5 (26%)	10 (21%)	6 (20%)	5 (33%)	0 (0%)	13 (17%)	15 (19%)
PAGI-QOL	n = 5	n = 21	n = 10	n = 7	n = 4	n = 33	n = 27
0–0.99	0 (0%)	1 (5%)	2 (20%)	0 (0%)	0 (0%)	2 (6%)	3 (11%)
1–1.99	2 (40%)	6 (29%)	4 (40%)	3 (43%)	3 (75%)	6 (18%)	12 (44%)
2–2.99	2 (40%)	6 (29%)	4 (40%)	4 (57%)	1 (25%)	12 (36%)	10 (37%)
3–3.99	0 (0%)	6 (29%)	0 (0%)	0 (0%)	0 (0%)	9 (27%)	2 (7%)
4–4.99	1 (20%)	2 (10%)	0 (0%)	0 (0%)	0 (0%)	4 (12%)	0 (0%)

n represents the number of patients with that HRSN.

Three hundred twenty-one patients were evaluated for the relationship of HRSN, to GCSI; 109 patients were evaluated for the relationship of HRSN, to PAGI-QOL.

#### Relationship between HRSN and gastroparesis symptom severity

**Figure 1:**
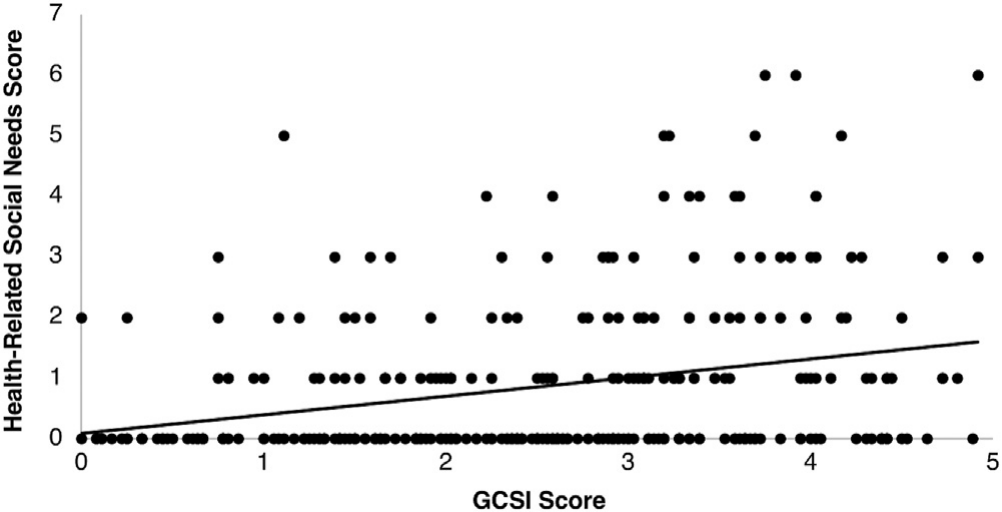
Association between HRSN and severity of gastroparesis via GCSI scores. Correlation of HRSN scores against GCSI (n = 321) to assess relationship between HRSN and severity of gastroparetic symptoms. Pearson’s r = 0.280, *P* < .001. y = 0.3075x + 0.0856.

Overall, HRSN score had a direct correlation to GCSI total symptoms score (r = 0.284, *P* < .05) (Figure 1). Patients with any HRSN, except for utility needs and interpersonal safety, tend to have higher GCSI score, evident in Table 2 which shows that the prevalence of patients with HRSN increased as GCSI score ranges increased from 0–0.99 to 3–3.99, . Interestingly, the prevalence of patients with HRSN decreased in all domains as GCSI score ranges increased from 3–3.99 to 4–4.99. For example, in the mental health domain, only 2 patients (2%) had GCSI scores ranging 0–0.99. The prevalence of patients with a need in mental health increased from 10 patients (12%) to 35 patients (43%) as GCSI score ranges increased from 1–1.99 to 3–3.99 while the prevalence of patients decreased to 15 patients (19%) as GCSI score ranges increased to 4–4.99. For example, the prevalence of patients with a need in mental health increased from 2 patients to 35 patients as GCSI score range increased from 0–0.99 to 3–3.99 but the prevalence decreased to 15 patients as GCSI score range increased to 4–4.99.

The average GCSI score was significantly greater (*P* < .05) in patients with HRSN in the domains of food insecurity, transportation needs, utility needs, financial strain, and mental health (Table 3). The average GCSI score was greater in patients with HRSN in living situation and interpersonal safety but the results were not statistically significant (*P* > .05).

**Table 3:** Average GCSI and PAGI-QOL Scores Between Patients With and Without HRSN in Each Domain

GCSI/PAGI-QOL scores	Average GCSI/PAGI-QOL without HRSN ± SD (n)	Average GCSI/PAGI-QOL with HRSN ± SD (n)	Difference	*P* value
GCSI				
Living situation	2.50 ± 1.19 (302)	3.00 ± 1.22 (19)	0.50	.099
Food insecurity	2.44 ± 1.18 (274)	3.05 ± 1.12 (47)	0.61	**.001**
Transportation needs	2.47 ± 1.19 (291)	3.10 ± 1.05 (30)	0.63	**.004**
Utility needs	2.49 ± 1.18 (306)	3.37 ± 1.19 (15)	0.88	**.013**
Interpersonal safety	2.53 ± 1.20 (314)	2.56 ± 1.17 (7)	0.03	.935
Financial strain	2.41 ± 1.18 (246)	2.90 ± 1.17 (75)	0.49	**.002**
Mental health	2.33 ± 1.20 (240)	3.13 ± 0.95 (81)	0.80	**.001**
PAGI-QOL				
Living situation	3.42 ± 1.15 (104)	2.54 ± 1.15 (5)	0.88	.163
Food insecurity	3.56 ± 1.10 (88)	2.61 ± 1.12 (21)	0.95	**.002**
Transportation needs	3.54 ± 1.06 (99)	1.76 ± 0.86 (10)	1.79	**.001**
Utility needs	3.46 ± 1.14 (102)	2.15 ± 0.51 (7)	1.31	**.001**
Interpersonal safety	3.43 ± 1.15 (105)	1.99 ± 0.44 (4)	1.44	**.002**
Financial strain	3.66 ± 1.08 (76)	2.72 ± 1.07 (33)	0.95	**<.0001**
Mental health	3.86 ± 0.79 (82)	1.92 ± 0.85 (27)	1.94	**<.0001**

Bold indicate statistically significant values.

SD, standard deviation.

#### Relationship between HRSN and health-related quality of life

**Figure 2:**
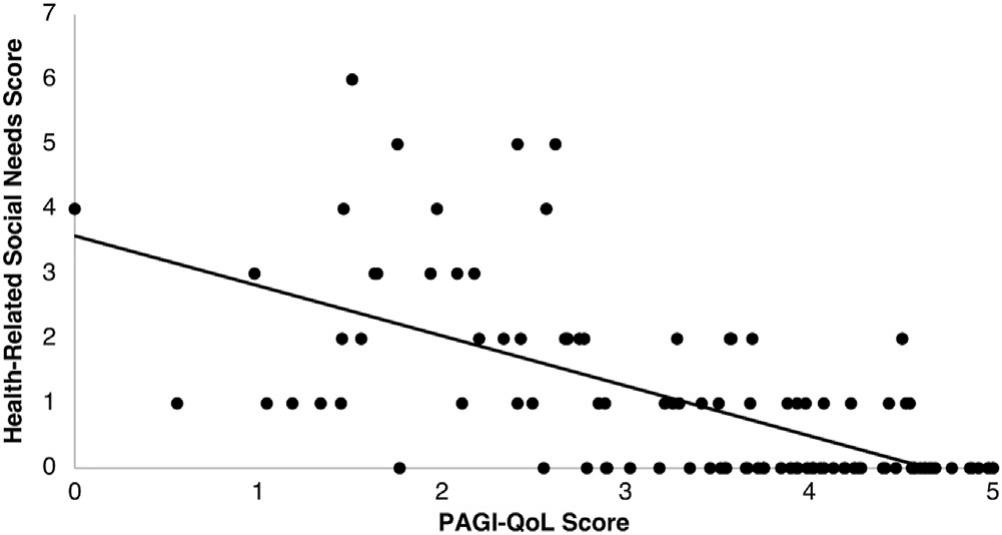
Association between HRSN and quality of life via PAGI-QOL scores. Correlation of HRSN scores against PAGI-QOL (n = 109) to assess relationship between HRSN and quality of life. Pearson’s r = −0.6499, *P* < .001. y = −0.771x + 3.5858.

In the 109 patients who completed the PAGI-QOL questionnaire, the number of HRSN was inversely correlated to the PAGI-QOL score (r = −0.650, *P* < .05) (Figure 2). The prevalence of patients with HRSN in food insecurity, transportation needs, utility needs, interpersonal safety, financial strain, and mental health increased as PAGI-QOL score ranges decreased from 4–4.99 to 2–2.99 (Table 2). On the other hand, the prevalence of patients with needs in any HRSN increased as PAGI-QOL score ranges increased from 0–0.99 to 1–1.99. For example, in the financial strain domain, the prevalence increased from 4 patients to 12 patients as PAGI-QOL score range decreased from 4–4.99 to 2–2.99. However, the prevalence decreased to 2 patients as PAGI-QOL score range decreased to 0–0.99.

Average PAGI-QOL scores were significantly lower (*P* < .05) in patients with needs in mental health, financial strain, transportation needs, food insecurity, utility needs, and interpersonal safety (Table 3). Average PAGI-QOL score was lower in patients with needs in their living situation but the results were not significant (*P* > .05).

### Analysis of Patients with None or Only 1 HRSN

There is an overlap among HRSN with a number of patients (22%) having multiple HRSN which might impact associating individual HRSN domains with gastroparesis scores. To address this, we took additional analyses to focus on patients with either none or only 1 HRSN, thereby removing those with multiple HRSNs.

#### Relationship between HRSN and gastroparesis symptom severity

Two hundred forty-nine of the 321 total patients who completed HRSN and GCSI questionnaires had either none or 1 HRSN (Table 4). Financial strain (n = 33) and mental health (n = 18) were the most commonly seen domains in patients with only 1 HRSN. The average GCSI score was significantly greater in patients with HRSN in the mental health domain (2.87 vs 2.32; *P* = .012). Although not statistically significant (primarily due to low numbers of patients with the HRSN), the average GCSI score was greater in patients with needs in living situation, transportation needs, utility needs, and financial strain.

**Table 4:** Average GCSI and PAGI-QoL Scores Between Patients With and Without HRSN in Each Domain for Patients With None or 1 HRSN

GCSI/PAGI-QOL scores	Average GCSI/ PAGI-QOL without HRSN ± SD (n)	Average GCSI/PAGI-QOL with HRSN ± SD (n)	Difference	*P* value
GCSI				
Living situation	2.39 ± 1.18 (246)	2.56 ± 1.26 (3)	0.17	.8060
Food insecurity	2.39 ± 1.18 (244)	2.13 ± 0.65 (5)	0.25	.6340
Transportation needs	2.37 ± 1.17 (245)	3.17 ± 1.64 (4)	0.80	.1807
Utility needs	2.38 ± 1.18 (247)	3.49 ± 1.32 (2)	1.11	.1876
Interpersonal safety	2.39 ± 1.18 (248)	1.67 (1)	0.72	n/a
Financial strain	2.37 ± 1.19 (231)	2.59 ± 1.14 (18)	0.22	.4376
Mental health	2.32 ± 1.19 (216)	2.87 ± 1.05 (33)	0.55	**.0125**
PAGI-QOL				
Living situation	3.74 ± 0.99 (80)	4.44 (1)	0.70	n/a
Food insecurity	3.75 ± 1.00 (78)	3.90 ± 0.37 (3)	0.16	.7863
Transportation needs	3.75 ± 0.99 (81)	n/a (0)	n/a	n/a
Utility needs	3.75 ± 0.99 (81)	n/a (0)	n/a	n/a
Interpersonal safety	3.75 ± 0.99 (81)	n/a (0)	n/a	n/a
Financial strain	3.78 ± 1.02 (71)	3.56 ± 0.75 (10)	0.21	.5234
Mental health	3.99 ± 0.70 (71)	2.05 ± 1.09 (10)	1.94	**<.0001**

Bold indicate statistically significant values.

n represents the number of patients with that HRSN.

Two hundred forty-nine of 321 total patients who completed HRSN, and GCSI, questionnaires with either none or one HRSN, included in this table.

Eighty-one of 109 total patients who completed HRSN, and PAGI-QOL, questionnaires with either none or 1 HRSN, included in this table.

#### Relationship between HRSN and health-related quality of life

Eighty-one of the 109 total patients who completed the PAGI-QOL questionnaire had either no HRSN or only one HRSN. Patients expressed needs in the domains of mental health (10 patients), financial strain (10 patients), food insecurity (3 patients), and living situation (1 patient) (Table 4). Average PAGI-QOL scores were significantly lower (*P* < .05) in patients with HRSN only in the mental health domain (2.05 vs 3.99; *P* < .01). Although not statistically significant, PAGI-QOL scores were lower in patients with HRSN in financial strain.

## Discussion

Out of the 321 patients who participated in this study, 138 patients (43%) had at least 1 HRSN and 72 patients (22%) had at least 2 HRSN. The greatest number of patients had needs in mental health, financial strain, and food insecurity. We found that there is a direct correlation between severity of gastroparesis symptoms and the number of HRSNs patients have. We also found that there was an inverse correlation between quality of life and the number of HRSNs that patients have. This demonstrates the potential impact of HRSN in patients with gastroparesis.

Patients’ social needs may affect several disease processes. Studies of cardiovascular disease show increased risk of fatal incident coronary heart disease and nonfatal myocardial infarctions were associated with patients who had poorer social determinants of health.^6^ Minority race/ethnic groups were found to have a higher prevalence of obesity, active inflammatory bowel disease, and comorbid conditions such as diabetes, hypertension, and lung disease.^12^ In this study, we took a step further to understand the impact of social needs on our patients by looking at types of HRSN and by analyzing which HRSN have the greatest impact on patients’ severity of gastroparesis and quality of life. Patients with needs in mental health, in particular, had significantly more severe symptoms of gastroparesis and had significantly lower quality of life. Two other HRSN domains, financial strain and food insecurity, were also common in our patients.

Our study finding mental health was an important HRSN in gastroparesis highlights the importance of addressing any underlying mental health issues patients may present with in the clinic. Woodhouse et al^13^ in 2017 conducted a literature review of studies addressing the relationship between gastroparesis and outcome measures of anxiety, depression, or quality of life. The literature review revealed that gastroparesis is associated with significant psychological distress and poor quality of life, aligning with the findings in our study. In another study by Hasler et al^14^ in 2010, higher depression and anxiety scores were associated with gastroparesis severity but did not have any associations with etiology or degree of gastric retention further emphasizing the importance of addressing underlying mental health issues when managing gastroparesis.

Studies on HRSN are important to assist healthcare professionals by providing guidance on how to help patients improve their health outcomes and quality of life while living with certain diseases. For example, Hong and Mainous^15^ in 2020 developed a validated health risk assessment tool for cardiovascular disease based on 3 SDOH domains (socioeconomic status, food/lifestyle, and health care resources). Kangovi et al^16^ in 2017 created a community health worker intervention program called IMPaCT (Individualized Management for Patient-Centered Targets) to address upstream socioeconomic and behavioral barriers and measured prespecified primary outcomes of several chronic diseases including diabetes, hypertension, and smoking.

Several limitations exist in this study. For example, our HRSN questionnaire was less extensive than the screening tool created by CMS. Our HRSN questionnaire included 7 domains (living situation, food insecurity, transportation needs, utility needs, interpersonal safety, financial strain, and mental health), while the screening tool created by CMS originally had 5 core domains (living situation, food insecurity, transportation problems, utility help needs, and interpersonal safety) in addition to 8 supplemental domains (financial strain, employment, family and community support, education, physical activity, substance use, mental health, and disabilities). Therefore, several potentially key domains were not examined in this study. In addition, part of our analysis was comparing GCSI and PAGI-QOL scores of patients who had either no HRSN or only 1 need in any individual HRSN domain which reduced the number of patients. This limits our study given that often patients with any HRSN present with multiple. It is rare to see patients with a need in living situation without a need for financial strain. However, this analysis was necessary to avoid patients with overlapping domains and to get a clearer picture of how specific domains affect severity of gastroparesis and quality of life. Finally, only 109 patients out of 321 total patients completed PAGI-QOL, as it was added after the study had started. Given the small sample size of 109, the results for the part of our study that explored the relationship between PAGI-QOL and HRSN are less compelling. Although our study showed that there are correlations between HRSN and severity of gastroparesis symptoms and quality of life, the study does not necessarily show whether there is a causal relationship between these factors; this would require improving patients’ HRSN and studying the effects on their symptoms of gastroparesis and quality of life.

## Conclusion

In summary, this study was able to identify patients with HRSN by using a HRSN screening tool. A large number (43%) of patients with gastroparesis had at least one HRSN. HRSN were associated with more severe gastroparesis symptoms and worse quality of life. The most common HRSN were mental health, financial strain, and food insecurity Recognizing and addressing HRSN in patients with gastroparesis may improve symptoms and quality of life.
